# Non-Unique Solutions of Magnetohydrodynamic Stagnation Flow of a Nanofluid towards a Shrinking Sheet Using the Solar Radiation Effect

**DOI:** 10.3390/mi14030565

**Published:** 2023-02-27

**Authors:** Sumayyah Alabdulhadi, Anuar Ishak, Iskandar Waini, Sameh E. Ahmed

**Affiliations:** 1 Department of Mathematical Sciences, Faculty of Science and Technology, Universiti Kebangsaan Malaysia (UKM), Bangi 43600, Malaysia; 2 Department of Mathematics, Faculty of Science, Qassim University, Qassim 52571, Saudi Arabia; 3 Fakulti Teknologi Kejuruteraan Mekanikal dan Pembuatan, Universiti Teknikal Malaysia Melaka, Durian Tunggal, Melaka 76100, Malaysia; 4 Department of Mathematics, Faculty of Science, King Khaild University, Abha 62529, Saudi Arabia; 5 Department of Mathematics, Faculty of Science, South Valley University, Qena 83523, Egypt

**Keywords:** stagnation-point flow, nanofluid, solar energy, dual solutions, stability analysis

## Abstract

This study aims to investigate the magnetohydrodynamic flow induced by a moving surface in a nanofluid and the occurrence of suction and solar radiation effects using the Buongiorno model. The numerical findings are obtained using MATLAB software. The effects of various governing parameters on the rates of heat and mass transfer along with the nanoparticles concentration and temperature profiles are elucidated graphically. Non-unique solutions are discovered for a specific variation of the shrinking strength. The temporal stability analysis shows that only one of them is stable as time passes. Furthermore, raising the Brownian motion parameter reduces both the local Sherwood number and the local Nusselt number for both solutions. It is also observed that increasing the thermophoresis parameter reduces the rate of heat transfer, whereas the opposite trend is observed for the rate of mass transfer.

## 1. Introduction

Renewable energy is becoming more popular as a way to minimize reliance on finite fossil fuel supplies and alleviate the effects of climate change. Since it transforms solar energy directly into heat and electricity without emitting greenhouse gases, solar energy has been established as the greatest and most inexpensive renewable energy source [1]. Studies during the past few years on solar energy have indicated that nanofluids play a major role in enhancing efficiency in solar systems due to their outstanding ability to transfer heat [2,3,4,5]. Choi and Eastman [6] pioneered the discovery of nanofluids by combining nanometer-sized particles with conventional fluids. Nanofluids are superior coolants in transportation, nuclear reactors, lubricants, thermal storage, domestic refrigerators, optical devices, and cancer therapeutics since nanoparticles have more thermal conductivity compared to base fluid [7,8,9,10]. Two models have been proposed by Tiwari and Das [11] and Buongiorno [12] for investigating the nanofluid properties. It is notable to mention that the model by Buongiorno is a non-homogeneous two-phase model with a slip velocity for the base fluid as well as nanoparticles that are not equal to zero. This model has several slip mechanisms: Brownian diffusion, gravity, thermophoresis, diffusiophoresis, fluid drainage, the Magnus effect, and inertia. Meanwhile, the Tiwari and Das model is a homogeneous single-phase model that investigates the influence of the solid volume fraction of nanoparticles on nanofluid behavior. Multiple scholars have utilized these two models to examine the characteristics of flow and heat transmission of a nanofluid under different physical situations [13,14,15,16].

Magnetohydrodynamic (MHD) is a field of study that investigates the magnetic field effect on electrically conductive fluids. In recent times, the study of magnetohydrodynamic has attracted numerous scholars given its wide implementations in engineering and industry, such as nuclear reactors, crystal growth, optical fiber filters, optical grafting, liquid metals, petroleum industries, and metallurgical processes [17]. Lund et al. [18] investigated the MHD mixed convection flow over a shrinking/stretching sheet in a nanofluid with a viscous dissipation effect. The results revealed that various solutions were produced; nevertheless, the first solution was determined to be the most stable. With the occurrence of injection/suction parameters, Naramgari and Sulochana [19] investigated the chemical reaction and thermal radiation effects on the constant magnetohydrodynamic flow of a nanofluid driven by a permeable shrinking/stretching surface. They discovered that as thermophoresis parameters and Brownian motion enhance, the nanoparticle concentration decreases while the mass transfer rate increases. The magnetic field influence on the free convection flow in a corrugated container loaded with nanofluids was investigated by Ahmed and Rashed [20]. The topic of hydromagnetic flow of third-grade nanofluid caused by a nonlinear deformable sheet under the impact of activation energy and chemical reactions was considered by Hayat et al. [21]. Afterward, Ayub et al. [22] used the Lorentz force impact to study the Carreau nanofluid flow, taking into account the influence of infinite shear rate viscosity. Ayub et al. [23] researched the infinite shearing rate influence on MHD Carreau nanofluid flow induced by a cylindrical channel. They discovered that increasing the impact of an inclined magnetic dipole tends to raise the energy while decreasing the velocity.

The stagnation flow demonstrates the behavior of fluid movement close to the stagnation point. Considering its wide applications in both scientific and industrial areas, the research of stagnation point flow is of great significance. In addition, Hiemenz [24] was the earliest to investigate the flow past a flat sheet at a two-dimensional stagnation point. Moreover, Dzulkifli et al. [25] examine the heat transfer rate and unstable stagnation-point flow towards an exponentially stretching surface in nanofluids, considering the velocity slip effect. Yashkun et al. [26] investigated the features of the stagnation point flow and heat transfer across a shrinking/stretching sheet with a suction effect. Subsequently, Kamal et al. [27] investigated the mixed effects of injection/suction, chemical reaction, and magnetic field on the flow in a nanofluid caused by a permeable shrinking/stretching surface. Finally, the constant two-dimensional magnetohydrodynamic stagnation flow of a nanofluid via a shrinking sheet was considered by Aladdin et al. [28]. It was inferred during the examination that when the magnetic field was enhanced, the fluid velocity, heat, and mass transmission rates increased. Still, the rate of heat and mass transfer is reduced when the thermophoresis parameter is enhanced. Since then, a great number of studies on the stagnation point flow have been completed in many respects in fluid dynamics, for instance [29,30,31,32,33,34].

Existing literature confirmed that no investigation has been done before on the magnetohydrodynamic flow of a nanofluid towards a shrinking sheet in the presence of solar radiation and suction using the Buongiorno model. To fill the above knowledge gap, a mathematical formulation of the current study is created with reference to the study conducted by Ghasemi and Hatami [35]. This research is different from that considered by Ghasemi and Hatami [35], where we consider a shrinking sheet with suction effects. The impact of the various physical parameters on the Sherwood number and the Nusselt number, along with the nanoparticles’ concentration and temperature profiles, are depicted in the tabular form and graph. Besides, the emergence of non-unique solutions as well as their stability are discussed.

## 2. Mathematical Formulation

**Figure 1:**
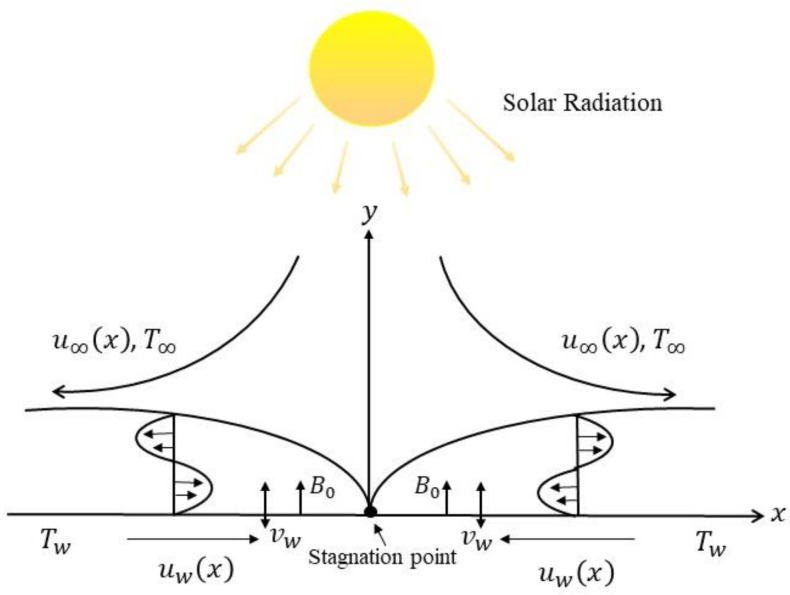
Diagram of the present study.

The two-dimensional magnetohydrodynamic flow towards a stretching/shrinking surface with solar radiation and suction effects is considered, as illustrated in Figure 1. It is assumed that $u_{w}\left(x\right)=ax$ is the surface velocity and $u_{\infty }\left(x\right)=bx$ is the free stream velocity, where a and b are the positive constants. It is noted that a>0 is for stretching and a<0 is for shrinking. Moreover, the ambient fluid temperature and the convective surface temperature are signified as $T_{\infty }$ and $T_{f}$, respectively. A magnetic field of uniform strength $B_{0}$ is implemented parallel to the direction of the flow. Given the previous assumptions, the governing equations can be written as [35]:

(1) $$\frac{\partial u}{\partial x}+\frac{\partial v}{\partial y}=0$$

(2) $$u\frac{\partial u}{\partial x}+v\frac{\partial u}{\partial y}=u_{\infty }\frac{du_{\infty }}{dx}+v_{f}\frac{\partial^{2}u}{\partial y^{2}}-\frac{\sigma_{e}B_{0}^{2}}{\rho_{f}}\left(u-u_{\infty }\right)$$

subject to the boundary conditions:

(3) $$u=u_{W}\left(x\right)=ax,\;v=v_{w}\left(x\right)\;at\;y=0$$

$$u\to u_{\infty }\left(x\right)=bx\;as\;y\to \infty$$

Here u and v denote the component of velocity in the x and y axes, respectively, $v_{w}\left(x\right)$ implies the mass flux velocity, $\nu_{f}$ represents the kinematic viscosity, $\sigma_{e}$ signifies the electrical conductivity, and $B_{0}$ indicates constant magnetic field in the y direction. The following similarity transformation is employed to solve Equations (1)–(3):

(4) $$\eta =\sqrt{\frac{b}{\nu_{f}}}y,\;\psi =\sqrt{\nu_{f}b}xf\left(\eta \right),\;u=bxf^{\prime }\left(\eta \right),\;v=-\sqrt{b\nu_{f}}f\left(\eta \right)$$

By utilizing the similarity transformation (4), the continuity Equation (1) is satisfied, while Equations (2) and (3) reduce to

(5) $$f^{\prime \prime \prime }+ff^{\prime \prime }-{f^{\prime }}^{2}+1-M\left(f^{\prime }-1\right)=0$$

(6) $$f\left(0\right)=s,\;f^{\prime }\left(0\right)=\varepsilon ,\;f^{\prime }\left(+\infty \right)\to 1$$

where $M=\sigma_{e}B_{0}^{2}/\rho_{f}b$ indicates the magnetic parameter, ε=a/b symbolizes the stretching/shrinking parameter, and $s=-v_{w}/\sqrt{a\nu_{f}}$ signifies the suction/injection parameter. On the other hand, the energy and concentration equations are respectively given by

(7) $$u\frac{\partial T}{\partial x}+v\frac{\partial T}{\partial y}=\alpha \frac{\partial^{2}T}{\partial y^{2}}-\frac{1}{{\left(\rho c\right)}_{f}}\left(\frac{\partial q_{r}}{\partial y}\right)+\tau D_{B}\left[\frac{\partial T}{\partial y}\frac{\partial C}{\partial y}+\frac{D_{T}}{T_{\infty }}{\left(\frac{\partial T}{\partial y}\right)}^{2}\right]$$

(8) $$u\frac{\partial C}{\partial x}+v\frac{\partial C}{\partial y}=D_{B}\frac{\partial^{2}C}{\partial y^{2}}+\frac{D_{T}}{T_{\infty }}\frac{\partial^{2}T}{\partial y^{2}}$$

where T represents the temperature, $C_{f}$ the allusion to the specific heat capacity, α implies the thermal diffusivity, and C denotes the nanoparticle concentration. The thermophoretic diffusion coefficient and the Brownian motion coefficient are signified by $D_{T}$ and $D_{B}$, respectively. Further, $q_{r}$ addresses the radiative heat flux and $\tau ={\left(\rho c\right)}_{p}/{\left(\rho c\right)}_{f}$ refers to the proportion between the nanoparticle effective heat capacity and the heat capacity of the fluid. By utilizing the Rosseland approximations, we get the radiative heat flux in the following form (see Raptis [36], Brewster [37], and Sparrow and Cess [38]).

(9) $$q_{r}=-\frac{4\sigma^{*}}{3k^{*}}\frac{\partial T^{4}}{\partial y}=-\frac{16\sigma^{*}}{3k^{*}}T^{3}\frac{\partial T}{\partial y}$$

In Equation (9), $\sigma^{*}$ represents Stefan-Boltzman constant, while $k^{*}$ denotes the mean absorption coefficient. By utilizing Equation (9), Equation (7) becomes

(10) $$u\frac{\partial T}{\partial x}+v\frac{\partial T}{\partial y}=\frac{\partial }{\partial y}\left[\left(\alpha +\frac{16\sigma^{*}T^{3}}{3{\left(\rho c\right)}_{f}k^{*}}\right)\frac{\partial T}{\partial y}\right]+\tau D_{B}\left[\frac{\partial T}{\partial y}\frac{\partial C}{\partial y}+\frac{D_{T}}{T_{\infty }}{\left(\frac{\partial T}{\partial y}\right)}^{2}\right]$$

The boundary constraints are expressed as

(11) $$-k\frac{\partial T}{\partial x}=h\left(T_{f}-T\right),\;C=C_{W}\;\;\mathrm{at}\;\;y=0\;\;T\to T_{\infty },\;C\to C_{\infty }\;\;\mathrm{as}\;\;y\to \infty$$

The non-dimensional temperature profile can be defined as $\theta \left(\eta \right)=(T-T_{\infty })/\left(T_{f}-T_{\infty }\right)$ with $T=T_{\infty }\left(1+\left(\theta_{w}-1\right)\theta \right)$ and $\theta_{w}=T_{f}/T_{\infty }$. The third term of Equation (10) can be expressed as $\alpha \frac{\partial }{\partial y}\left[\frac{\partial T}{\partial y}\left(1+R_{d}\left(1+\left(\theta_{w}-1\right)\theta^{3}\right)\right)\right]$ where $R_{d}=16\sigma^{*}T_{\infty }^{3}/3kk^{*}$ stands for the radiation parameter, and there is no thermal radiation effect when $R_{d}=0$. The last expression is then reduced to the following form:

(12) $$\frac{b\left(T_{f}-T_{\infty }\right)}{Pr}{\left[\left(1+R_{d}\left(1+\left(\theta_{w}-1\right)\theta^{3}\right)\right)\theta^{\prime }\right]}^{\prime }$$

where $Pr=\nu_{f}/\alpha$ denotes the Prandtl number.

Equations (8) and (10) are transformed to the following form:

(13) $$\frac{1}{Pr}{\left[\left(1+R_{d}\left(1+\left(\theta_{w}-1\right)\theta^{3}\right)\right)\theta^{\prime }\right]}^{\prime }+f\theta^{\prime }+N_{b}\theta^{\prime }\varphi^{\prime }+N_{t}{\theta^{\prime }}^{2}=0$$

(14) $$\varphi^{\prime \prime }+Le\;f\varphi^{\prime }+\frac{N_{t}}{N_{b}}\theta^{\prime \prime }=0$$

subject to the transformed boundary conditions:

(15) $$\theta^{\prime }\left(0\right)=-\left(Bi\right)\left[1-\theta \left(0\right)\right],\;\varphi \left(0\right)=1,\;\;\;\theta \left(+\infty \right)\to 0,\;\varphi \left(+\infty \right)\to 0$$

Here $\varphi \left(\eta \right)=c-c_{\infty }/c_{w}-c_{\infty }$ and Bi iis the Biot number, $N_{t}$ is the thermophoresis parameter; and $N_{b}$ is the Brownian motion parameter, which are expressed as $Bi=h/k\sqrt{\nu_{f}/\alpha }$,$\;N_{t}=\tau D_{T}\left(T_{w}-T_{\infty }\right)/T_{\infty }\nu_{f}$ and $N_{b}=\tau D_{B}\left(C_{w}-C_{\infty }\right)/\nu_{f}$, respectively. The surface heat and mass fluxes are expressed as:

$$q_{w}=-k{\left(\frac{\partial T}{\partial y}\right)}_{y=0}+{\left(q_{r}\right)}_{w}=-k\left(T_{f}-T_{\infty }\right)\sqrt{\frac{b}{\nu_{f}}}\left[1+R_{d}\theta_{w}^{3}\right]\theta^{\prime }\left(0\right)$$

(16) $$j_{w}=-D_{B}{\left(\frac{\partial C}{\partial y}\right)}_{y=0}=-D_{B}\left(C_{W}-C_{\infty }\right)\sqrt{\frac{b}{\nu_{f}}}\varphi^{\prime }\left(0\right)$$

By using (16), the local Nusselt number $Nu_{x}=xq_{w}/k\left(T_{f}-T_{\infty }\right)$ and the local Sherwood number $Sh_{x}=xj_{w}/D_{B}\left(C_{W}-C_{\infty }\right)$ become

(17) $$\frac{Nu_{x}}{\sqrt{Re_{x}}}=-\left[1+R_{d}\theta_{w}^{3}\right]\theta^{\prime }\left(0\right),\;\frac{Sh_{x}}{\sqrt{Re_{x}}}=-\varphi^{\prime }\left(0\right)$$

where $Re_{x}=b/\nu_{f}x^{2}$ is the local Reynolds number.

## 3. Stability Analysis

The numerical results demonstrate that there are two solutions for specific parameter values. As a result, it is necessary to look into the temporal stability of these solutions, to see which one is stable as time evolves. To perform a stability analysis of the dual solutions, as per Weidman et al. [39], this issue must be regarded in an unsteady form by introducing the new dimensionless time parameter τ. The unsteady case for the governing Equations (2), (8), and (10) is:

(18) $$\frac{\partial u}{\partial t}+u\frac{\partial u}{\partial x}+v\frac{\partial u}{\partial y}=u_{\infty }\frac{du_{\infty }}{dx}+v_{f}\frac{\partial^{2}u}{\partial y^{2}}-\frac{\sigma_{e}B_{0}^{2}}{\rho_{f}}\left(u-u_{\infty }\right)$$

(19) $$\frac{\partial u}{\partial t}+u\frac{\partial T}{\partial x}+v\frac{\partial T}{\partial y}=\frac{\partial }{\partial y}\left[\left(\alpha +\frac{16\sigma^{*}T^{3}}{3{\left(\rho C\right)}_{f}k^{*}}\right)\frac{\partial T}{\partial y}\right]+\tau D_{B}\left[\frac{\partial T}{\partial y}\frac{\partial C}{\partial y}+\frac{D_{T}}{T_{\infty }}{\left(\frac{\partial T}{\partial y}\right)}^{2}\right]$$

(20) $$\frac{\partial u}{\partial t}+u\frac{\partial C}{\partial x}+v\frac{\partial C}{\partial y}=D_{B}\frac{\partial^{2}C}{\partial y^{2}}+\frac{D_{T}}{T_{\infty }}\frac{\partial^{2}T}{\partial y^{2}}$$

where t refers to the time, the new similarity transformation is expressed as follows:

(21) $$\eta =\sqrt{\frac{b}{\nu_{f}}}y,\;\psi =\sqrt{\nu_{f}b}xf\left(\eta ,\tau \right),\;\theta \left(\eta ,\tau \right)=\frac{T-T_{\infty }}{T_{f}-T_{\infty }},\;\varphi \left(\eta ,\tau \right)=\frac{c-c_{\infty }}{c_{w}-c_{\infty }},\;\tau =bt$$

Applying Equation (21) into Equations (18)–(20), the following equations are obtained:

(22) $$\frac{\partial^{3}f}{\partial \eta^{3}}+f\frac{\partial^{2}f}{\partial \eta^{2}}-{\left(\frac{\partial f}{\partial \eta }\right)}^{2}+1-M\left(\frac{\partial f}{\partial \eta }-1\right)-\frac{\partial^{2}f}{\partial \eta \partial \tau }=0$$

(23) $$\frac{1}{Pr}{\left[\left(1+R_{d}\left(1+\left(\theta_{w}-1\right)\theta^{3}\right)\right)\frac{\partial \theta }{\partial \eta }\right]}^{\prime }+f\frac{\partial \theta }{\partial \eta }+N_{b}\frac{\partial \theta }{\partial \eta }\frac{\partial \varphi }{\partial \eta }+N_{t}{\left(\frac{\partial \theta }{\partial \eta }\right)}^{2}-\frac{\partial \theta }{\partial \tau }=0$$

(24) $$\frac{\partial^{2}\varphi }{\partial \eta^{2}}+Le\;f\frac{\partial \varphi }{\partial \eta }+\frac{N_{t}}{N_{b}}\frac{\partial^{2}\theta }{\partial \eta^{2}}-\frac{\partial \varphi }{\partial \tau }=0$$

subjected to

$$f\left(0,\tau \right)=0,\;\frac{\partial f}{\partial \eta }\left(0,\tau \right)=\varepsilon ,\;\frac{\partial \theta }{\partial \eta }\left(0,\tau \right)=-\left(Bi\right)\left[1-\theta \left(0\right)\right],\;\varphi \left(0,\tau \right)=1$$

(25) $$\frac{\partial f}{\partial \eta }\left(\eta ,\tau \right)=1,\;\theta \left(\eta ,\tau \right)=0,\;\varphi \left(\eta ,\tau \right)=0,\;\mathrm{as}\;\;\eta \to \infty =0$$

The following perturbations are introduced to investigate the flow stability in the long run, with $f=f_{0}\left(\eta \right)$, $\theta =\theta_{0}\left(\eta \right)$, and $\varphi =\varphi_{0}\left(\eta \right)$ which satisfy Equations (22)–(24) (see Weidman et al. [39]),

$$f\left(\eta .\tau \right)=f_{0}\left(\eta \right)+e^{-\gamma \tau }F\left(\eta \right)$$

(26) $$\theta \left(\eta .\tau \right)=\theta_{0}\left(\eta \right)+e^{-\gamma \tau }G\left(\eta \right)i$$

$$\varphi \left(\eta .\tau \right)=\varphi_{0}\left(\eta \right)+e^{-\gamma \tau }H\left(\eta \right)$$

where γ is an unknown eigenvalue. The linearized problems are obtained by incorporating Equation (26) into Equations (22)–(25):

(27) $$F^{\prime \prime \prime }+f_{0}F^{\prime \prime }-\left(2f_{0}^{\prime }+M-\gamma \right)F^{\prime }+f_{0}^{\prime \prime }F=0$$

(28) $$\frac{1}{Pr}{\left[\left(1+R_{d}\left(1+\left(\theta_{w}-1\right){\theta_{0}}^{3}\right)\right)G^{\prime }\right]}^{\prime }+\left(f_{0}+Nb\varphi_{0}^{\prime }+2Nt\theta_{0}^{\prime }\right)G^{\prime }+\theta_{0}^{\prime }F\;+Nb\theta_{0}^{\prime }H^{\prime }+\gamma G=0$$

(29) $$H^{\prime \prime }+Le\left(f_{0}H^{\prime }+\varphi_{0}^{\prime }F\right)+\frac{Nt}{Nb}G^{\prime \prime }+\gamma H=0$$

and are subjected to the boundary conditions

$$F\left(0\right)=0,\;F^{\prime }\left(0\right)=0,\;G^{\prime }\left(0\right),=Bi\;G\left(0\right),\;H\left(0\right)=0$$

(30) $$F^{\prime }\left(\eta \right)\to 0,\;G\left(\eta \right)\to 0,\;H\left(\eta \right)\to 0\;\;\;\;\;\mathrm{as}\;\;\;\;\eta \to \infty$$

Here, the least eigenvalue γ determines the stability of the steady flow solutions $f_{0}\left(\eta \right)$, $\theta_{0}\left(\eta \right)$ and $\varphi_{0}\left(\eta \right)$. Based on the study by Harris et al. [40], relaxation of the boundary condition on $F\left(\eta \right)$, $G\left(\eta \right)\;\mathrm{or}\;H\left(\eta \right)$ is required to obtain the possible eigenvalues. Thus, the condition $F_{0}^{\prime }\left(\eta \right)\to 0\;\mathrm{as}\;\eta \to \infty$ is relaxed, where the system (26)–(29) is solved along with the new boundary condition $F_{0}^{\prime \prime }=1$.

## 4. Findings and Discussion

**Figure 2:**
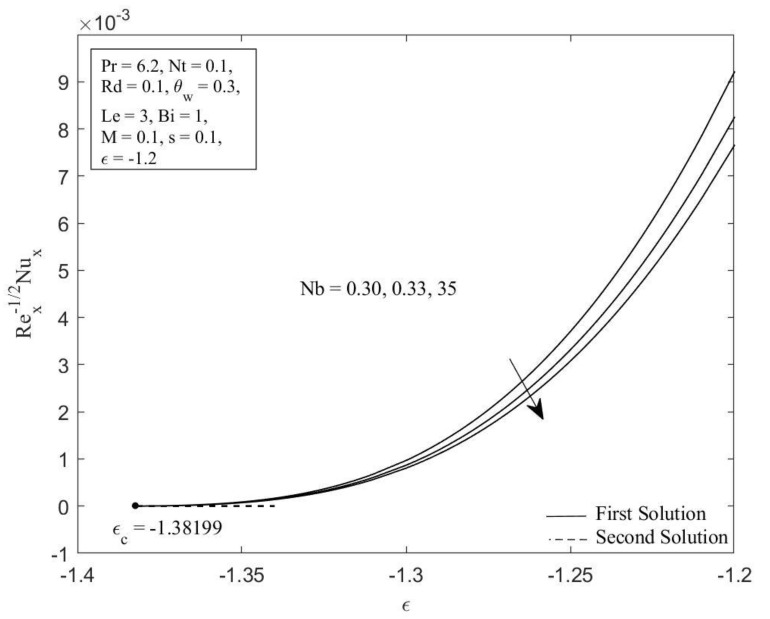
Local Nusselt number $Nu_{x}Re_{x}^{-1/2}$ against ε for diverse values of Nb.

**Figure 3:**
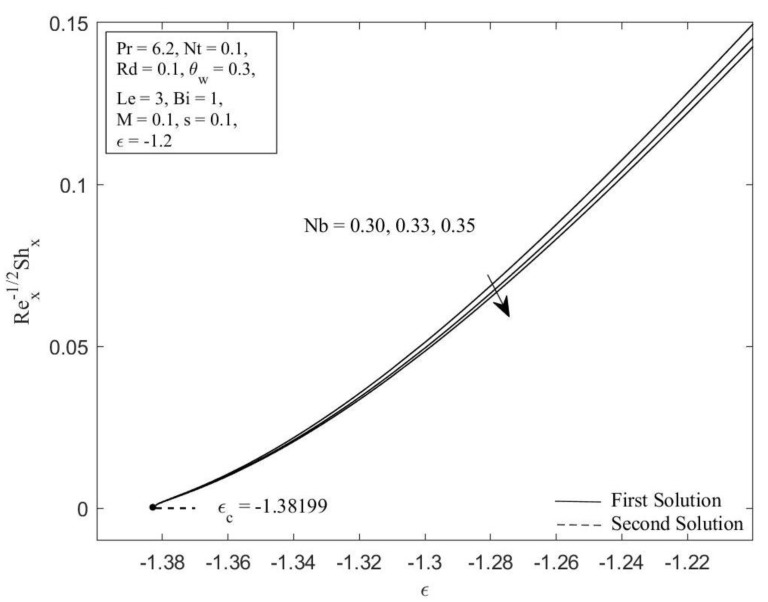
Local Sherwood number $Sh_{x}Re_{x}^{-1/2}$ against ε for diverse values of Nb.

**Figure 4:**
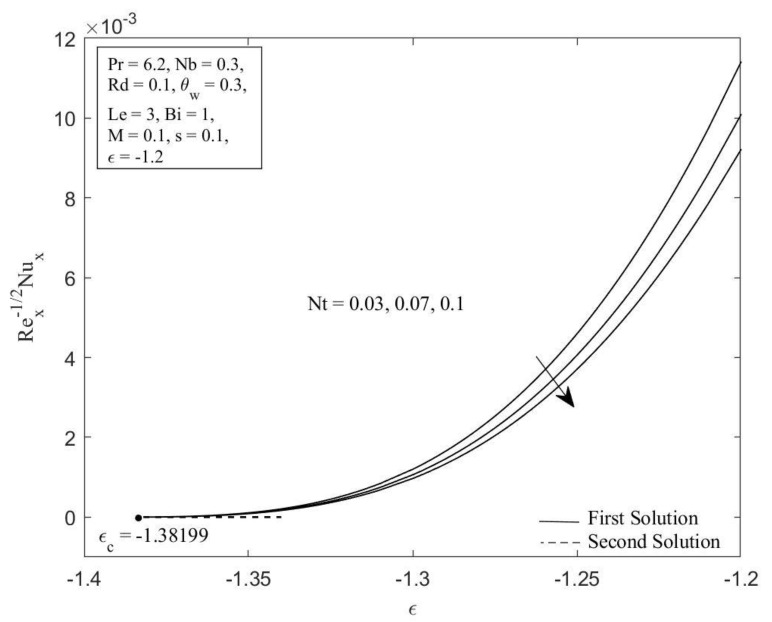
Local Nusselt number $Nu_{x}Re_{x}^{-1/2}$ against ε for diverse values of Nt.

**Figure 5:**
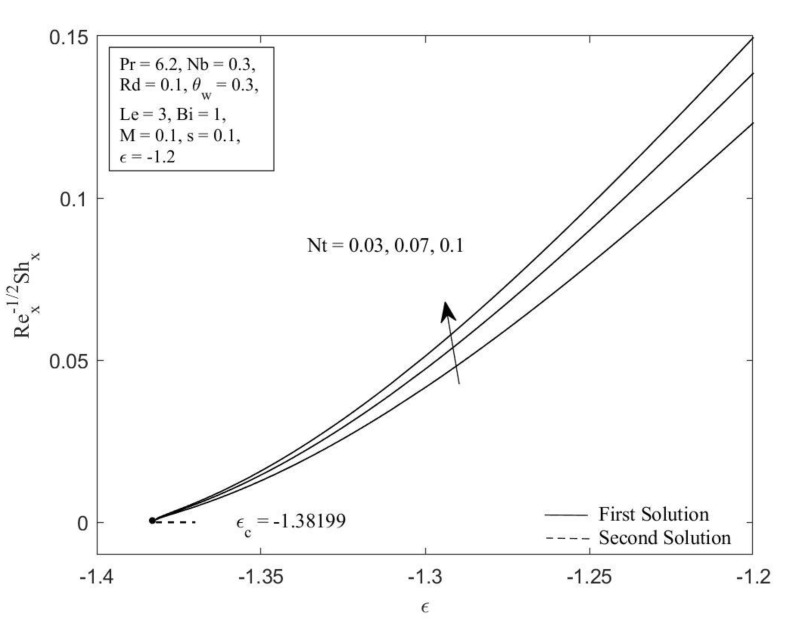
Local Sherwood number $Sh_{x}Re_{x}^{-1/2}$ against ε for diverse values of Nt.

**Figure 6:**
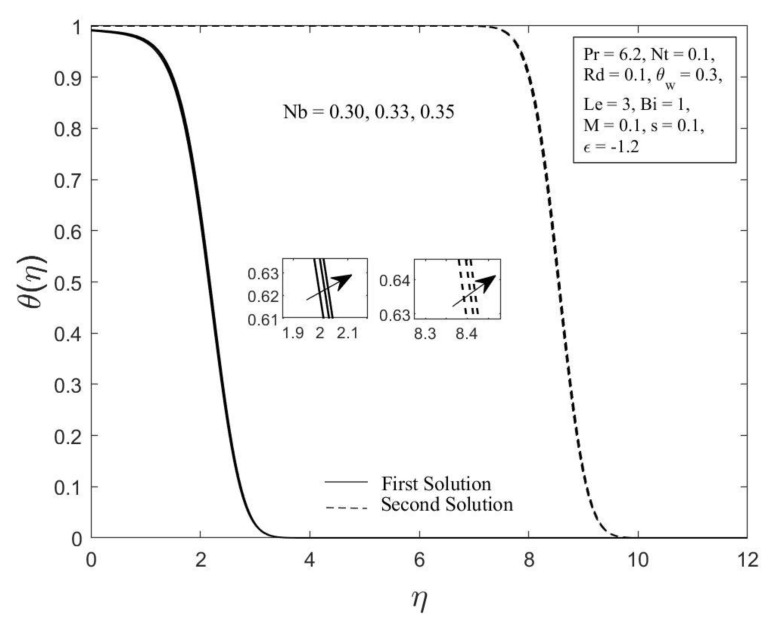
The temperature profile $\theta \left(\eta \right)$ for diverse values of Nb.

**Figure 7:**
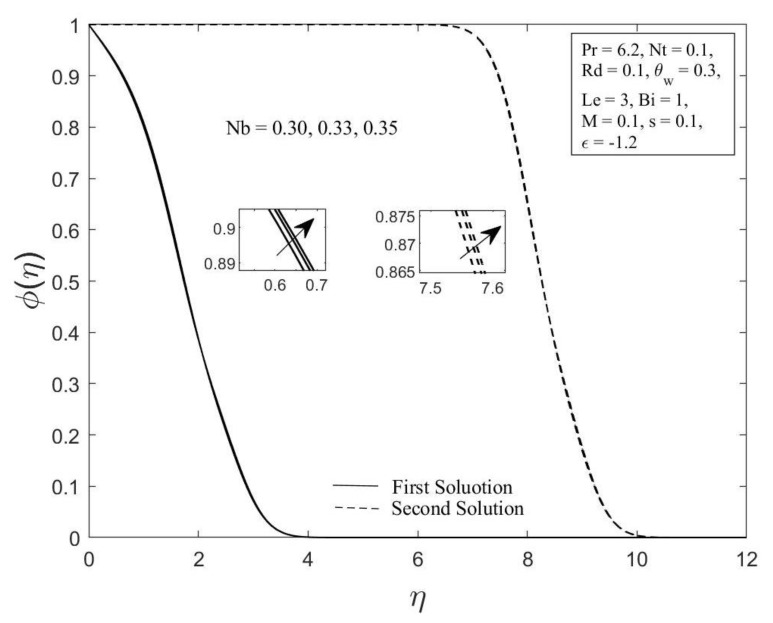
The concentration profile $\varphi \left(\eta \right)$ for diverse values of Nb.

**Figure 8:**
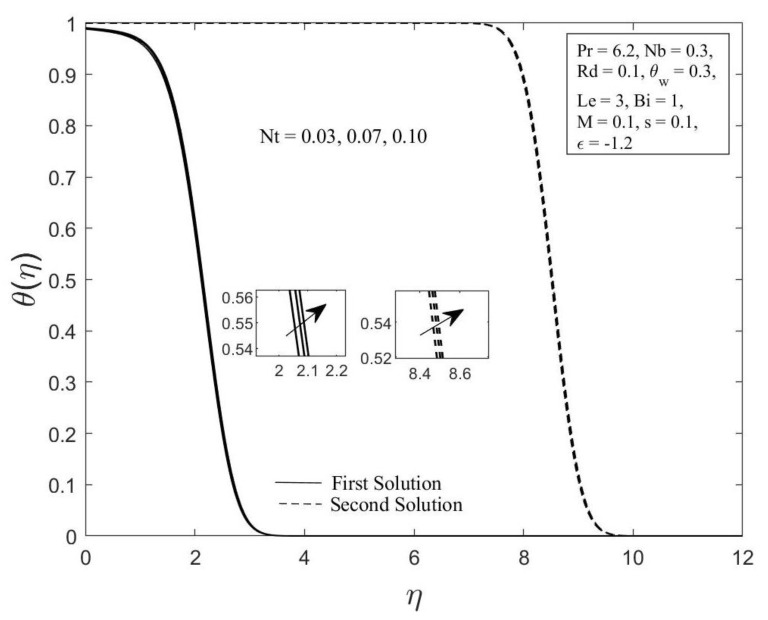
The temperature profile $\theta \left(\eta \right)$ for diverse values of Nt.

**Figure 9:**
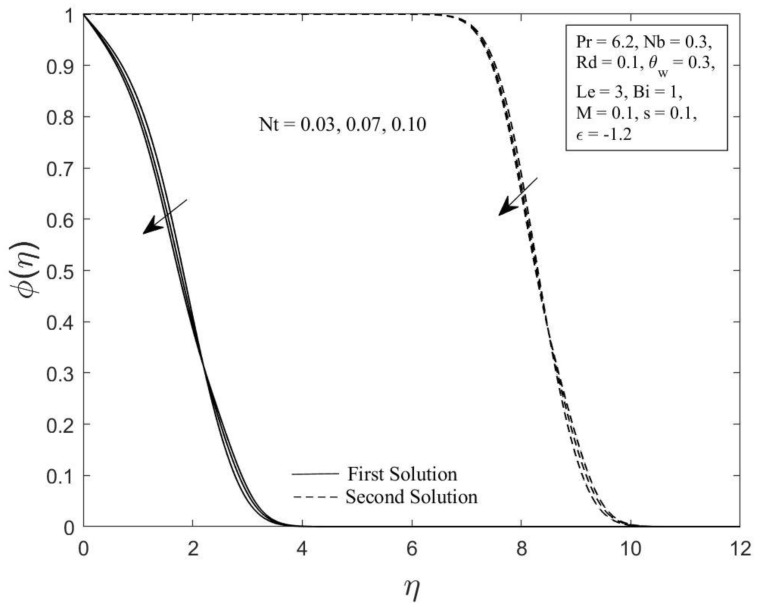
The concentration profile $\varphi \left(\eta \right)$ for diverse values of Nt.

MATLAB software (MATLAB R2022a, MathWorks, Inc., Natick, MA, USA) is employed to obtain the numerical solutions to the ordinary differential equations (ODEs) (5), (13), and (14) aligned with the boundary conditions (6) and (15). The impacts of the governing parameters on the local Nusselt number $Nu_{x}Re_{x}^{-1/2}$ (refers to the heat transfer rate) and local Sherwood number $Sh_{x}Re_{x}^{-1/2}$ (refers to the mass transfer rate), along with the temperature profile $\theta \left(\eta \right),$ and concentration profile $\varphi \left(\eta \right)$ are addressed graphically in Figure 2, Figure 3, Figure 4, Figure 5, Figure 6, Figure 7, Figure 8 and Figure 9. It is prominent to note that the dual solutions are discovered to exist at a specified level of the parameters towards the shrinking region $\left(\varepsilon >\varepsilon_{c}\right)$ and a unique solution is obtained when $\varepsilon =\varepsilon_{c}$. However, no solutions have been discovered for $\varepsilon <\varepsilon_{c}$, where $\varepsilon_{c}$ signifies the critical value of ε for which solutions exist. Moreover, a comparison of the heat transfer rate as well as the mass transfer rate is done with those recorded by Ghasemi and Hatami [35] and Khan and Pop [41], as shown in Table 1 and Table 2. The comparison shows excellent agreement. As a result, the accuracy of the findings reported in this study is assured.

Figure 2 and Figure 3 illustrate the Brownian motion parameter Nb impacts on the local Sherwood number as well as local Nusselt number with certain values of ε when Pr=6.2, Nt=0.1, Rd=0.1, $\theta_{w}=0.3$, Le=3, Bi=1, M=0.1, and s=0.1, respectively. Clearly, for both branches of solutions, increasing of Nb decreases the Sherwood number as well as the Nusselt number. This is caused as the Brownian motion parameter *Nb* is enhanced; the number of collisions between base fluid particles and nanoparticles increases, resulting in an increase of the thermal as well as the concentration boundary layer thickness. The local Sherwood and Nusselt numbers reduce as a result of this behavior. In Figure 4 and Figure 5, the graphical findings are displayed for the Nusselt number and Sherwood number at selected values of the thermophoresis parameter Nt against ε when Pr=6.2, Nb=0.3, Rd=0.1, $\theta_{w}=0.3$, Le=3, Bi=1, M=0.1, and s=0.1, respectively. In Figure 4, the reducing trends of the local Nusselt number in both solutions as the thermophoresis parameter Nt rises. This is due to the fact that the thermophoresis effect assists nanoparticles in transitioning from hot to cold regions, leading to a thicker thermal boundary layer and therefore reducing the heat transfer rate at the surface. Contrary to this, displays reverse behavior when the increment of the thermophoresis parameter Nt augments the local Sherwood number. The influence of thermophoresis will help the nanoparticles with high thermal conductivity penetrate further into the fluid, resulting in a thinner concentration boundary layer. Hence, the concentration gradient increases, which enhances the rate of mass transfer at the surface.

Moreover, the behavior of the temperature profile $\theta \left(\eta \right)$ as well as the concentration profile $\varphi \left(\eta \right)$ with a variety of Brownian motion parameter Nb, where Pr=6.2, Nt=0.1, Rd=0.1, $\theta_{w}=0.3$, Le=3, Bi=1, M=0.1, s=0.1, and ε=−1.2 are demonstrated in Figure 6 and Figure 7. The augmentation in the values of $\theta \left(\eta \right)$ and $\varphi \left(\eta \right)$ for both branches of solutions are observed with an upsurge in Nb values. This result is expected since the Brownian motion parameter causes the nanoparticles to agitate from a high concentration to a low concentration, driving more nanoparticles away from the shrinking surface. The concentration and thermal boundary layers will thicken as a result of this. The concentration and temperature gradients decrease as the thickness of the thermal and concentration boundary layers increase. As a result, the local Sherwood and Nusselt numbers declined. The temperature profile $\theta \left(\eta \right)$ as well as the concentration profile $\varphi \left(\eta \right)$ for chosen values of the thermophoresis parameter Nt where Pr=6.2, Nb=0.3, Rd=0.1, $\theta_{w}=0.3$, Le=3, Bi=1, M=0.1, s=0.1 and ε=−1.2 are depicted in Figure 8 and Figure 9, respectively. In both solutions, raising the thermophoresis parameter Nt boosts the temperature $\theta \left(\eta \right)$ while decreasing the concentration $\varphi \left(\eta \right)$. Additionally, the thermal boundary layer thickness rises as the thermophoresis parameter Nt rises, but the concentration boundary layer thickness drops. This culminates in a reduction in the temperature gradient and an elevation in the concentration gradient, resulting in a reduction in the heat transfer rate and an elevation of the mass transfer rate, as observed in Figure 4 and Figure 5. Note that the thermophoresis force acts opposite the gradient of the temperature in nanofluids as well as helping nanoparticles migrate from the hot surface to the cold ambient fluid.

**Figure 10:**
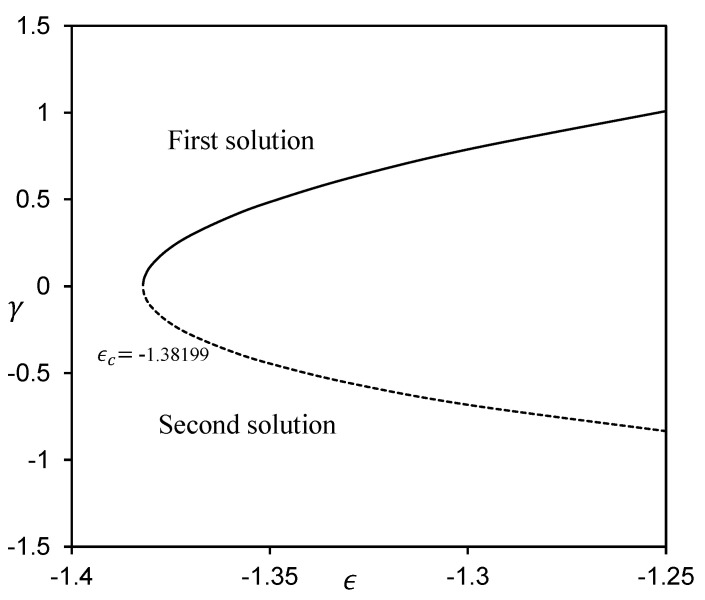
Smallest eigenvalues γ for different values of ε when $Pr=6.2,\;Nb=0.3,\;Nt=0.1,\;Rd=0.1,\;\theta_{w}=0.3,\;Le=3,\;Bi=1,\;M=0.1\;\mathrm{and}\;s=0.1$.

Lastly, by utilizing the bvp4c function in Matlab to solve Equations (27)–(30), an analysis of stability is executed. In Figure 10, the smallest eigenvalues γ for dual solutions when Pr=6.2, Nb=0.3, Nt=0.1, Rd=0.1, $\theta_{w}=0.3$, Le=3, Bi=1, M=0.1, and s=0.1 are presented for several values of ε. The eigenvalues play a crucial role in determining the dual solutions stability. Provided that the smallest eigenvalue γ is positive, this signifies that the solution appears stable and indicates that the flow has just slight disturbances that have no effect on the flow characteristics or physical appearance. Otherwise, a negative value for the smallest eigenvalues indicates that the solution is unstable, implying that the disturbance impacting the flow system is growing. Moreover, we may deduce from Figure 10 that the first solution is stable, whereas the other solution is not, as time evolves.

## 5. Conclusions

The mathematical model of the MHD flow of a nanofluid over a shrinking sheet under influenced by suction and solar radiation has been investigated. By employing the proper similarity variables, the governing PDEs are transformed to ODEs, which are subsequently numerically solved by employing the bvp4c solver in Matlab software. The primary outcomes obtained are summarized below:

- The outcomes deduce that the existence of dual (non-unique) solutions is provable for a given shrinking strength range (ε<−1).
- According to the temporal stability study, only the first solution is stable and, hence, physically significant.
- The Sherwood number as well as the Nusselt number decreases when the Brownian motion parameter upsurges.
- The rate of heat transfer reduces when the thermophoresis parameter Nt is elevated; however, the rate of mass transfer is found to increase.
  The concentration augments by incrementing the Brownian motion parameter Nb but reduces by elevating the thermophoresis parameter Nt.
- The influence of the Brownian motion parameter Nb and the thermophoresis parameter Nt on the temperature profile reveals that the thermal boundary layer thicknesses as well as the temperature increase for both solutions.

## Figures and Tables

**Table 1:** Comparison data for the values of $Nu_{x}Re_{x}^{-1/2}$ for various values of Nb when Pr=Le=10, Nt=0.1, and Rd=0.

Nb	Present Results	Ghasemi and Hatami [35]	Khan and Pop [41]
0.1	0.952377	0.9528	0.9524
0.2	0.505581	0.5057	0.5056
0.3	0.252156	0.2527	0.2522
0.4	0.119406	0.1196	0.1194
0.5	0.054253	0.0546	0.0543

**Table 2:** Comparison data for the values of $Sh_{x}Re_{x}^{-1/2}$ for various values of Nt when Pr=Le=10, Nt=0.1, and Rd=0.

Nt	Present Results	Ghasemi and Hatami [35]	Khan and Pop [41]
0.1	2.129393	2.1295	2.1294
0.2	2.274020	2.2744	2.2740
0.3	2.528636	2.5288	2.5286
0.4	2.795168	2.7955	2.7952
0.5	3.035140	3.0353	3.0351

## Data Availability

Not applicable.

## Nomenclature

a, b	constants
Bi	Biot number
$B_{0}$	uniform magnetic field (T)
C	nanoparticles concentration $\left({\mathrm{kgm}}^{-3}\right)$
$C_{f}$	skin friction coefficient
$C_{p}$	specific heat capacity $\left({\mathrm{Jkg}}^{-1}K^{-1}\right)$
$C_{w}$	wall nanoparticles concentration $\left({\mathrm{kgm}}^{-3}\right)$
$C_{\infty }$	ambient nanoparticle concentration $\left({\mathrm{kgm}}^{-3}\right)$
$D_{B}$	Brownian diffusion coefficient $\left(m^{2}s^{-1}\right)$
$D_{T}$	thermophoretic diffusion coefficient $\left({\mathrm{kgm}}^{-3}\right)$
f	dimensionless velocity
h	convective heat transfer coefficient
$j_{w}$	surface mass flux
k	thermal conductivity of the fluid $\left({\mathrm{Wm}}^{-1}K^{-1}\right)$
$k^{*}$	mean absorption coefficient
Le	Lewis number
M	magnetic parameter
Nb	Brownian motion parameter
Nt	thermophoresis parameter
$Nu_{x}$	local Nusselt number
Pr	Prandtl number
$q_{r}$	radiative heat flux $\left({\mathrm{Wm}}^{-2}\right)$
$q_{w}$	surface heat flux $\left({\mathrm{Wm}}^{-2}\right)$
Rd	thermal radiation
$Re_{x}$	local Reynolds number
$Sh_{x}$	local Sherwood number
s	constant mass flux
T	fluid temperature $\left(K\right)$
$T_{f}$	convective surface temperature $\left(K\right)$
$T_{\infty }$	ambient temperature $\left(K\right)$
t	time $\left(s\right)$
$u_{w}$	velocity of the stretching/shrinking sheet $\left({\mathrm{ms}}^{-1}\right)$
$u_{\infty }$	velocity of the free stream $\left({\mathrm{ms}}^{-1}\right)$
u,v	velocity component in the *x* and *y* directions $\left({\mathrm{ms}}^{-1}\right)$
$v_{w}$	velocity of the wall mass transfer $\left({\mathrm{ms}}^{-1}\right)$
x,y	Cartesian coordinates $\left(m\right)$
Greek symbols	
α	thermal diffusivity of the nanofluid $\left(m^{2}s^{-1}\right)$
ε	stretching/shrinking parameter
η	similarity variable
γ	eigenvalue
μ	dynamic viscosity $\left({\mathrm{kgm}}^{-1}s^{-1}\right)$
ν	kinematic viscosity $\left(m^{2}s^{-1}\right)$
φ	dimensionless nanoparticle volume fraction
ψ	stream function $\left(m^{2}s^{-1}\right)$
ρ	fluid density $\left({\mathrm{kgm}}^{-3}\right)$
${\left(\rho c\right)}_{f}$	heat capacity of the fluid $\left({\mathrm{JK}}^{-1}m^{-3}\right)$
${\left(\rho c\right)}_{p}$	heat capacity of the nanoparticles $\left({\mathrm{JK}}^{-1}m^{-3}\right)$
$\sigma_{e}$	electrical conductivity
$\sigma^{*}$	Stefan-Boltzman constant
τ	dimensionless time variable
θ	dimensionless temperature
Subscripts	
w	condition at the wall
∞	ambient condition
Superscript	
′	differentiation with respect to η

## References

[1] Hernandez R.R., Easter S.B., Murphy-Mariscal M.L., Maestre F.T., Tavassoli M., Allen E.B., Barrows C.W., Belnap J., Ochoa-Hueso R., Ravi S. (2014). Environmental impacts of utility-scale solar energy. Renew. Sustain. Energy Rev..

[2] Ladjevardi S.M., Asnaghi A., Izadkhast P.S., Kashani A.H. (2013). Applicability of graphite nanofluids in direct solar energy absorption. Sol. Energy.

[3] Kasaeian A., Eshghi A.T., Sameti M. (2015). A review on the applications of nanofluids in solar energy systems. Renew. Sustain. Energy Rev..

[4] Mahian O., Kianifar A., Kalogirou S.A., Pop I., Wongwises S. (2013). A review of the applications of nanofluids in solar energy. Int. J. Heat Mass Transf..

[5] Khanafer K., Vafai K. (2018). A review on the applications of nanofluids in solar energy field. Renew. Energy.

[6] Choi S.U.S., Eastman J.A. (1995). Enhancing Thermal Conductivity of Fluids with Nanoparticles.

[7] Saidur R., Leong K.Y., Mohammed H.A. (2011). A review on applications and challenges of nanofluids. Renew. Sustain. Energy Rev..

[8] Bhogare R.A., Kothawale B.S. (2013). A review on applications and challenges of nanofluids as coolant in automobile radiator. Int. J. Sci. Res. Publ..

[9] Gupta M., Singh V., Kumar R., Said Z. (2017). A review on thermophysical properties of nanofluids and heat transfer applications. Renew. Sustain. Energy Rev..

[10] Wong K.V., De Leon O. (2010). Applications of nanofluids: Current and future. Adv. Mech. Eng..

[11] Tiwari R.K., Das M.K. (2007). Heat transfer augmentation in a two-sided lid-driven differentially heated square cavity utilizing nanofluids. Int. J. Heat Mass Transf..

[12] Buongiorno J. (2006). Convective transport in nanofluids. J. Heat Transfer.

[13] Wang F., Sajid T., Ayub A., Sabir Z., Bhatti S., Shah N.A., Sadat R., Ali M.R. (2022). Melting and entropy generation of infinite shear rate viscosity Carreau model over Riga plate with erratic thickness: A numerical Keller Box approach. Waves Random Complex Media.

[14] El Din S.M., Darvesh A., Ayub A., Sajid T., Jamshed W., Eid M.R., Hussain S.M., Sánchez-Chero M., Ancca S.M., Ramírez Cerna J.M. (2022). Quadratic multiple regression model and spectral relaxation approach for carreau nanofluid inclined magnetized dipole along stagnation point geometry. Sci. Rep..

[15] Waini I., Ishak A., Pop I. (2021). Dufour and Soret effects on Al_2_O_3_-water nanofluid flow over a moving thin needle: Tiwari and Das model. Int. J. Numer. Methods Heat Fluid Flow.

[16] Soid S.K., Ishak A., Pop I. (2017). Unsteady MHD flow and heat transfer over a shrinking sheet with ohmic heating. Chin. J. Phys..

[17] Daniel Y.S., Aziz Z.A., Ismail Z., Salah F. (2019). Thermal radiation on unsteady electrical MHD flow of nanofluid over stretching sheet with chemical reaction. J. King Saud Univ..

[18] Lund L.A., Omar Z., Khan I. (2019). Quadruple solutions of mixed convection flow of magnetohydrodynamic nanofluid over exponentially vertical shrinking and stretching surfaces: Stability analysis. Comput. Methods Programs Biomed..

[19] Naramgari S., Sulochana C. (2016). MHD flow over a permeable stretching/shrinking sheet of a nanofluid with suction/injection. Alex. Eng. J..

[20] Ahmed S.E., Rashed Z.Z. (2019). MHD natural convection in a heat generating porous medium-filled wavy enclosures using Buongiorno’s nanofluid model. Case Stud. Therm. Eng..

[21] Hayat T., Riaz R., Aziz A., Alsaedi A. (2020). Influence of Arrhenius activation energy in MHD flow of third grade nanofluid over a nonlinear stretching surface with convective heat and mass conditions. Phys. A Stat. Mech. Its Appl..

[22] Ayub A., Sabir Z., Shah S.Z.H., Mahmoud S.R., Algarni A., Sadat R., Ali M.R. (2022). Aspects of infinite shear rate viscosity and heat transport of magnetized Carreau nanofluid. Eur. Phys. J. Plus.

[23] Ayub A., Sajid T., Jamshed W., Zamora W.R.M., More L.A.V., Talledo L.M.G., Rodríguez Ortega de Peña N.I., Hussain S.M., Hafeez M.B., Krawczuk M. (2022). Activation energy and inclination magnetic dipole influences on Carreau nanofluid flowing via cylindrical channel with an infinite shearing rate. Appl. Sci..

[24] Hiemenz K. (1911). Die Grenzschicht an einem in den gleichförmigen Flüssigkeitsstrom eingetauchten geraden Kreiszylinder. Dinglers Polytech. J..

[25] Dzulkifli N.F., Bachok N., Pop I., Yacob N.A., Arifin N.M., Rosali H. (2018). Unsteady Stagnation-Point flow and Heat Transfer Over an Exponential Stretching Sheet in Copper-Water Nanofluid with Slip Velocity Effect. Journal of Physics: Conference Series.

[26] Yashkun U., Zaimi K., Bakar N.A.A., Ferdows M. (2020). Nanofluid stagnation-point flow using Tiwari and Das model over a stretching/shrinking sheet with suction and slip effects. J. Adv. Res. Fluid Mech. Therm. Sci..

[27] Kamal F., Zaimi K., Ishak A., Pop I. (2019). Stability analysis of MHD stagnation-point flow towards a permeable stretching/shrinking sheet in a nanofluid with chemical reactions effect. Sains Malays..

[28] Aladdin N.A.L., Bachok N., Anuar N.S. (2020). MHD stagnation point flow in nanofluid over shrinking surface using Buongiorno’s model: A stability analysis. J. Adv. Res. Fluid Mech. Therm. Sci..

[29] Ishak A., Lok Y.Y., Pop I. (2010). Stagnation-point flow over a shrinking sheet in a micropolar fluid. Chem. Eng. Commun..

[30] Gupta S., Kumar D., Singh J. (2018). MHD mixed convective stagnation point flow and heat transfer of an incompressible nanofluid over an inclined stretching sheet with chemical reaction and radiation. Int. J. Heat Mass Transf..

[31] Kumar R.K., Varma S.V.K. (2019). Stagnation point flow of thermally radiative and dissipative MHD nanofluid over a stretching sheet filled with porous medium and suction. Songklanakarin J. Sci. Technol..

[32] Lund L.A., Omar Z., Khan I., Baleanu D., Nisar K.S. (2020). Convective effect on magnetohydrodynamic (MHD) stagnation point flow of Casson fluid over a vertical exponentially stretching/shrinking surface: Triple solutions. Symmetry.

[33] Abdul Halim N., Mohd Noor N.F. (2021). Mixed convection flow of Powell–Eyring nanofluid near a stagnation point along a vertical stretching sheet. Mathematics.

[34] Patil V.S., Patil A.B., Ganesh S., Humane P.P., Patil N.S. (2021). Unsteady MHD flow of a nano powell-eyring fluid near stagnation point past a convectively heated stretching sheet in the existence of chemical reaction with thermal radiation. Mater. Today Proc..

[35] Ghasemi S.E., Hatami M. (2021). Solar radiation effects on MHD stagnation point flow and heat transfer of a nanofluid over a stretching sheet. Case Stud. Therm. Eng..

[36] Raptis A. (1998). Radiation and free convection flow through a porous medium. Int. Commun. Heat Mass Transf..

[37] Brewster M.Q. (1992). Thermal Radiative Transfer and Properties.

[38] Sparrow E.M., Cess R.D. (2018). Radiation Heat Transfer: Augmented Edition.

[39] Weidman P.D., Kubitschek D.G., Davis A.M.J. (2006). The effect of transpiration on self-similar boundary layer flow over moving surfaces. Int. J. Eng. Sci..

[40] Harris S.D., Ingham D.B., Pop I. (2009). Mixed convection boundary-layer flow near the stagnation point on a vertical surface in a porous medium: Brinkman model with slip. Transp. Porous Media.

[41] Khan W.A., Pop I. (2010). Boundary-layer flow of a nanofluid past a stretching sheet. Int. J. Heat. Mass Transf..

